# ActiveCA: Time use data from the general social survey of Canada to study active travel

**DOI:** 10.1177/23998083251374724

**Published:** 2025-08-25

**Authors:** Bruno Dias dos Santos, Mahdis Moghadasi, Antonio Páez

**Affiliations:** School of Earth, Environment and Society, 3710 McMaster University, Hamilton, ON, Canada

**Keywords:** active, mobility, walking, cycling, travel time, time-use

## Abstract

This paper describes {ActiveCA}, an open data product with Canadian time use data. {ActiveCA} is an R data package that contains analysis-ready data related to active travel spanning almost 40 years, extracted from Cycles 2 (1986), 7 (1992), 12 (1998), 19 (2005), 24 (2010), 29 (2015), and 34 (2022) of the Time Use Survey (TUS) from the General Social Survey (GSS). Active travel episodes are characterized by mode, with walking being part of every cycle and bicycling starting in 1992. The attributes of active trips are the types of locations of origins and destinations, the duration of trips, and episode weights for expanding the trips to population-wide estimates. Based on the year of the survey, a variety of locations are coded. In earlier cycles, these include home, work or school, and other’s home, whereas in later cycles these are augmented with locations such as grocery stores, restaurants, outdoor destinations, and others. The geographical resolution includes the province and whether the episode was in an urban or rural setting.

## Introduction

The objective of this paper is to introduce {ActiveCA}, an open data product with data from all Time Use Survey (TUS) cycles of the Canadian General Social Surveys (GSSs). Open data products (ODPs) are the outcome of a process that transforms raw data (open or not) into analysis-ready data, following a transparent process in which all stages of development follow open principles (Arribas-Bel et al., 2021). ODPs, while still open, differ from general open data in their degree of ease of access, their heightened usability, and potentially the value they add to the raw data.

{ActiveCA} provides analysis-ready data concerning active travel in Canada spanning a period of almost 40 years, obtained from the TUS cycles of the Canadian GSS. The GSS program is designed to provide cross-sectional data on topics of interest to improve the well-being of Canadians. As part of this program, every five to 7 years the survey is done on the topic of time use. Concretely, {ActiveCA} covers Cycles 2 (1986), 7 (1992), 12 (1998), 19 (2005), 24 (2010), 29 (2015), and 34 (2022) of the TUS. Time use data in these surveys is coded using a very fine grain, from time spent in chores, leisure, and sleeping, to time spent working or at school. These surveys have proved valuable in investigations of mobility and quality of life (Spinney et al., 2009), the relationship between active travel and transit use (Lachapelle and Pinto, 2016), and travel behavior and time poverty (Kim et al., 2024), to name but a few examples.

Using PUMFs from the TUS, we extracted all data necessary to characterize active travel in Canada—specifically, episodes in which the activity involved moving between an origin and a destination by walking or cycling. Although Statistics Canada provides PUMFs and accompanying documentation for the GSS program (see Canada, 2024), accessing and preparing these files for analysis is not a straightforward task due to their size and complexity. The process of extracting information of interest from the source files is time-consuming, tedious, and challenging and/or prone to error due to the expertise required to work with these files. To create {ActiveCA} we selected, labeled, and processed the TUS cycles to make them ready for analysis.

{ActiveCA} is distributed as an R package with a number of data objects and their documentation. R packages contain code, data, and documentation in a standardized format that can be installed by R users via a software repository, such as CRAN (Comprehensive R Archive Network) or GitHub, which makes them an adroit medium to distribute analysis-ready data.

Given the level of interest in active travel (e.g., McCurdy et al., 2023), reducing the barriers to using data contained in rich, but difficult to access and use surveys, such as TUS, is a worthy endeavor that can only improve data-driven decisions in transportation, urban, and health policy. The rest of this paper discusses the sources of data, and the process implemented to retrieve and package them. Then, we show some selected examples of analysis to whet the imagination of potential users. This ODP provides not only data that are easy to use but also all the code and documentation that make this a reproducible research project. In summary, {ActiveCA} aims to implement and inspire the best principles of open spatial sciences (Brunsdon and Comber, 2021; Páez, 2021).

## The Time Use Survey (TUS) collection

Canada (2024) conducts GSS surveys to obtain data on social trends to track changes in Canadians’ living conditions and well-being over time. TUS are used to understand how Canadian residents spend and manage their time, and what factors contribute to their happiness and stress. The GSS program was created in 1985, and is serialized to provide a collection of annual, representative cross-sectional surveys.

The topics of the survey cycle every few years to cover topics that include family, health, social identity, and every five to 7 years time use. The first Canadian TUS done as part of the GSS program was conducted in 1986, and the most recent was completed in 2022. These surveys (Canada, 2022) collect data on respondents’ participation and time spent on a wide range of everyday activities using a 24-h retrospective diary, with information on the location of these activities (e.g., at home, at work, etc.) and, for non-personal activities, the people who were present with the respondent at the time of the activity. In addition, time-use surveys also cover topics related to leisure time, work-life balance, health, commuting, culture and sports, and many others.

TUS allows researchers to identify the origin and destination of trips, travel times and modes of transport used, providing a valuable dataset for analyzing active travel behavior. It also provides the empirical basis for tools used in transportation analysis, such as the development of impedance functions for accessibility analysis, a measure of the ease with which people can reach destinations and opportunities (Hansen, 1959). The Canadian TUS is unique at the national level in collecting detailed information on travel behavior. Its consistent application across survey cycles enables the identification of long-term trends, with some questions present in the questionnaires since the first application of the survey.

Most respondents to the 2022 TUS completed it online, reflecting Statistics Canada’s effort to adapt to technological changes and growing time demands by offering greater flexibility and convenience (Canada, 2022). While such methodological changes may affect data comparability over time, it is not possible to determine whether observed differences result from actual population changes or shifts in data collection methods. Despite rigorous efforts to ensure data quality, the use of electronic questionnaires may have influenced estimates. Statistics Canada assessed the impact of collection mode on a limited set of key questions, constrained by sample size. Importantly, none of the variables used in this research are in the 2022 PUMF User Guide as unsuitable for trend analysis.

Until 2022, Statistics Canada employed a telephone-based sampling frame, which was replaced by a dwelling-based frame in the most recent cycle. Each survey cycle spans a 12-month period, typically from July to the following July. The target population includes all Canadians aged 15 and over, excluding residents of the Yukon, Northwest Territories, and Nunavut, full-time institutional residents, and individuals living on Indigenous reserves.

The survey encompasses both rural and urban areas, including metropolitan and non-metropolitan regions, to ensure a diverse and representative sample. For sampling, the 10 provinces were divided into geographic stratas. Several Census Metropolitan Areas (CMAs)—such as St. John’s, Halifax, Saint John, Montreal, Quebec City, Toronto, Ottawa, Hamilton, Winnipeg, Regina, Saskatoon, Calgary, Edmonton, and Vancouver—were treated as separate strata. Additional strata grouped other CMAs within Quebec, Ontario, and British Columbia, as well as non-CMA areas within each province.

The Public Use Microdata Files (PUMFs) are released by Statistics Canada in two files: a main file and an episode file. The files are linked by keys that identify households, individuals, and episodes (i.e., activities) conducted by individuals. We discuss these files in more detail in the following section.

### The main file

The main file of the TUS compiles a large array of aggregated data, summarizing the answers to the questionnaire that describe households and individuals, as well as derived variables that summarize the respondents’ use of time use across different activities, locations, and social interactions. This file documents the time and duration that respondents allocate to each activity and location. The main file provides an overview of daily routines and social dynamics, not focusing on individual activity episodes. Additionally, this file categorizes activities into bigger groups and subcategories, facilitating the data’s analytical utility with additional metrics such as total transit time, time spent with household members, and counts of activities and episodes.

Table 1 shows the first ten rows and first six variables of the TUS PUMF 2015 main file (Cycle 29). Each row in the table correspond to a survey respondent, while the columns refer the following information: record identification (PUMFID), the person’s weight (WGHT_PER), the month the survey data was collected (SURVMNTH), the respondent’s age group (AGEGR10), the respondent’s sex (SEX), and the respondent’s marital status (MARSTAT).

**Table 1.** Visualization of the first ten lines and first six columns of the Main file of the 2015 GSS.

PUMFID	WGHT_PER	SURVMNTH	AGEGR10	SEX	MARSTAT
10,000	616.6740	7	5	1	5
10,001	8516.6140	7	5	1	1
10,002	371.7520	1	4	2	1
10,003	1019.3135	3	6	2	5
10,004	1916.0708	9	2	1	6
10,005	1952.2015	4	1	1	6
10,006	5761.5528	8	1	1	6
10,007	466.0426	6	5	2	3
10,008	2479.2991	2	2	2	1
10,009	1436.1641	8	6	1	3

Note. PUMFID: record identification. WGHT_PER: person weight. SURVMNTH: survey month of data collection. AGEGR10: age group of the respondent. SEX: sex of the respondent. MARSTAT: marital status of the respondent.

The main file of the 2015 GSS surveys includes a total of 17,390 respondents, representing 29,766,399 individuals and 848 variables. For discrete variables, Statistics Canada has assigned specific codes to the possible values, with each code accompanied by a label. For instance, in the case of the variable SURVMNTH, a value of 1 means January 2016, 2 means February 2016, 3 corresponds to March 2016, and so on.

As shown in Table 1, the variables are not labeled. Additionally, the format of the tables (comma-separated values) does not allow for the specification of variable types (whether a variable is continuous or discrete), which can lead to mistakes analysts who have limited experience working with PUMFs.

### The episode file

The episode is a much bigger file that records detailed data for each activity episode reported by respondents. Each episode represents a single activity and its duration, and the sum of all episodes throughout the day adds up to 24 h. Each entry in this file includes the start and end times of the activity, the duration, location, and accompanying social context, informing when and where activities occurred and with whom. The focus of the episode file is not on the characteristics of the respondents, but on the characteristics of the activities, and the data are structured around the numerous activity instances that compose a day of the respondent. Although respondent-specific characteristics are not included within the episode file, it is possible to link the main file and the episode file by using a key present in both the main and episodes files.

Similar to Table 1, which displayed an example of the main file structure, Table 2 presents the first seven episodes for the record identification number 10,041 and some variables from the TUS PUMF 2015 episode file (Cycle 29). Each row in the table corresponds to an episode associated with the specified record identification (PUMFID = 10041), including the episode’s weight (WGHT_EPI), episode number (EPINO), activity code (TUI_01), episode duration (DURATION), and episode location (LOCATION).

**Table 2.** Visualization of the first seven episodes of the record number 10,041.

PUMFID	EPINO	WGHT_EPI	TUI_01	DURATION	LOCATION
10,041	1	1353.818	1	210	300
10,041	2	1353.818	2	40	300
10,041	3	1353.818	27	15	300
10,041	4	1353.818	7	15	315
10,041	5	1353.818	9	180	300
10,041	6	1353.818	6	15	300
10,041	7	1353.818	18	120	300

Note. PUMFID: record identification. EPINO: episode number. WGHT_EPI: episode’s weight. TUI_01: activity code. DURATION: episode’s duration. LOCATION: episode’s location.

In total, the episode file of the 2015 GSS surveys contains 274,108 records, representing 461,837,622 episodes and 527 variables detailing the episodes. Similar to the main file, Statistics Canada has created codes for the discrete variables, with each value corresponding to a label.

In the case illustrated in Table 2, this respondent began the diary description by sleeping at home (TUI_01 = 1 and LOCATION = 300) for 210 minutes, followed by 40 minutes of personal hygiene (TUI_01 = 2). The respondent then spent 15 minutes on personal care activities, such as getting ready for school, supervising homework, reading, playing, reprimanding, or providing educational or emotional support, as indicated by TUI_01 = 27. Next, they recorded a travel episode, walking for 15 min (TUI_01 = 7 and LOCATION = 315), where both the origin and destination were their home. Such trips, where the journey starts and finishes at home, can be classified as recreational or leisure trips. Next, the respondent spent 3 h searching for a job (TUI_01 = 9), took a 15-min lunch break (TUI_01 = 6), and then cleaned the house (TUI_01 = 18) for 2 h. Table 2 displays only six variables out of the 527 available. As shown, since the dataset does not label the variable values, decoding them can be both time-consuming and challenging.

## Data process

**Figure 1.**
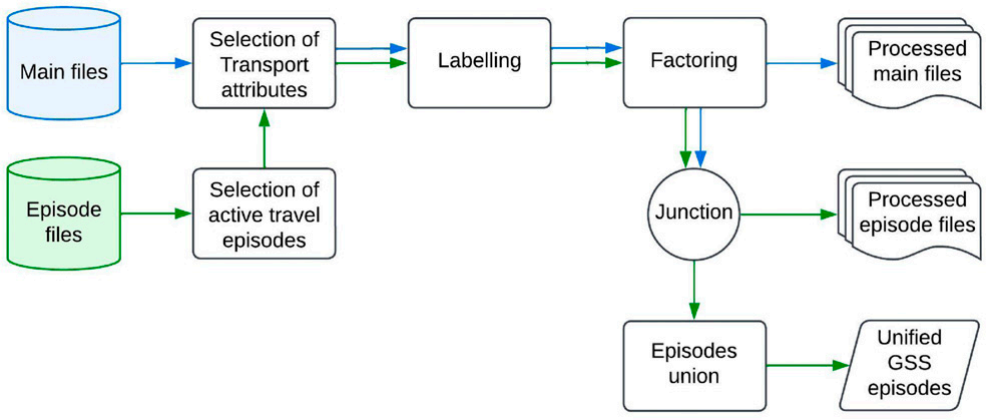
Diagram with the processes applied to the main (blue arrows) and episode files (green arrows) to obtain the ActiveCA datasets.

Figure 1 presents a diagram illustrating the processes applied to the main and episode files to create the {ActiveCA} datasets. For each TUS cycle, we reviewed the episode files to identify movement episodes involving walking or cycling. This allowed us to also select the activities immediately before and after the movement episode, helping to infer the trip’s purpose and determine its origin and destination. Active trips were identified by their corresponding activity codes, accounting for variations across survey cycles.

Next, we labeled the coded variables with their appropriate descriptions, classifying each origin and destination, mode of travel, and time spent in the active trip. Categorical variables were converted into factor variables, and ordinal variables were defined as ordered factors.

For the main files, we selected socioeconomic variables to help profile individuals engaged in active travel episodes. These included key indicators such as age group, sex, marital status, and number of children, among others. As with the episode files, we labeled and factored the coded variables. Using the appropriate identifiers, we joined the episode and main files to determine the province and the type of population center—whether it is a CMA, a Census Agglomeration (CA), or a non-CMA/CA area.

As output, the {ActiveCA} package provides processed datasets of walking and cycling episodes by year, a unified dataset that contains all walking and cycling episodes across TUS cycles, and processed main files (not unified, as socioeconomic and demographic variables and their categories differ across TUS cycles).

## {ActiveCA} data sets

This section presents some potential applications of the {ActiveCA} R package. In fact, we expect that the application of this package to extend beyond our pre-imagined range of uses. The installation instruction and also some examples of application of the {ActiveCA} R package are available in the vignettes, available in the GitHub repository.

### Active episodes

Table 3 displays the total number of records processed for main and episode files. For the main files, a total of 101,667 records were processed, referring to all records from the TUSs from 1986 to 2022 that together represents more of 181,526,641 respondents. It also presents the total cases of active trips episodes identified. In total 23,513 records with register of active travel activity. Together, these records account for 44,316,110 episodes.

**Table 3.** Total number and weighted sum of records processed.

	Main		Episode	
Year	Count	Weighted	Count_ep	Weighted_ep
2022	12,336	32,136,802	1765	6,041,032
2015	17,390	29,766,399	3496	6,634,387
2010	15,390	28,075,610	4615	8,516,753
2005	19,597	26,095,819	5866	7,583,838
1998	10,749	24,260,137	1789	3,606,987
1992	9,815	21,294,313	1635	3,691,918
1986	16,390	19,897,562	4347	8,241,196

Table 4 shows the first ten rows and first six variables of the TUS PUMF 2015 main file (Cycle 29), displayed in 1 before our processing. Table 5 presents the walking episodes for the record identification number 10041 from the TUS PUMF 2015 episode file (Cycle 29), previously displayed in Table 2. Only the unique active travel episode appears in Table 5 since the records were filtered to select cases with walking or cycling episodes. For both cases, Tables 4 and 5 contain labeled variables, facilitating the interpretation of the data.

**Table 4.** Visualization of the first ten lines and first six columns of the 2015 TUS Main File.

PUMFID	WGHT_PER	SURVMNTH	AGEGR10	SEX	MARSTAT
10,000	616.6740	July	55 to 64 years	Male	Divorced
10,001	8516.6140	July	55 to 64 years	Male	Married
10,002	371.7520	January	45 to 54 years	Female	Married
10,003	1019.3135	March	65 to 74 years	Female	Divorced
10,004	1916.0708	September	25 to 34 years	Male	Single, never married
10,005	1952.2015	April	15 to 24 years	Male	Single, never married
10,006	5761.5528	August	15 to 24 years	Male	Single, never married
10,007	466.0426	June	55 to 64 years	Female	Widowed
10,008	2479.2991	February	25 to 34 years	Female	Married
10,009	1436.1641	August	65 to 74 years	Male	Widowed

Note. PUMFID: record identification. WGHT_PER: person weight. SURVMNTH: survey month of data collection. AGEGR10: age group of the respondent. SEX: sex of the respondent. MARSTAT: marital status of the respondent.

**Table 5.** Visualization of the active travel episode for the record number 10,041 of the 2015 GSS survey.

PUMFID	WGHT_EPI	Activity	Duration	Origin	Destination	Mode
10,041	1353.818	Transport to or from activity	15	Home	Home	Walking

Note. PUMFID: record identification. EPINO: episode number. WGHT_EPI: episode’s weight. TUI_01: activity code. DURATION: episode’s duration. LOCATION: episode’s location.

### Descriptive statistics

Considering all TUS analyzed, we identified 23,513 episodes that recorded active travel episodes, with trip duration ranging from 0 to 900 min, to twelve different destinations. {ActiveCA} includes all these episodes ready for analysis. Table 6 presents descriptive statistics on walking and cycling trips between 1986 and 2022, with measures of trip duration in minutes. The 1986 survey did not include bicycle trips.

**Table 6.** Descriptive statistics of the duration (in minutes) of episodes with active transport records.

		Year						
Mode	Statistic	1986	1992	1998	2005	2010	2015	2022
Walking	Maximum	660	300	255	515	480	900	480
	Mean	21	21	12	12	13	18	19
	Median	15	10	5	10	10	10	15
	Minimum	1	1	1	0	0	5	5
	Standard deviation	31	25	17	16	17	27	24
Cycling	Maximum		240	90	180	153	120	150
	Mean		28	24	20	19	25	40
	Median		15	15	15	10	20	30
	Minimum		5	2	1	1	5	5
	Standard deviation		36	18	18	23	20	20

Table 6 shows that, until 2022 the median values for walking trips were 10 min, increasing to 15 min in the last survey. In the case of cycling trips, the duration fluctuated over the years, ranging from 10 to 30 min. The table also highlights very high maximum values, particularly for walking trips, with recorded episodes exceeding 4 hours in all cases.

**Figure 2.**
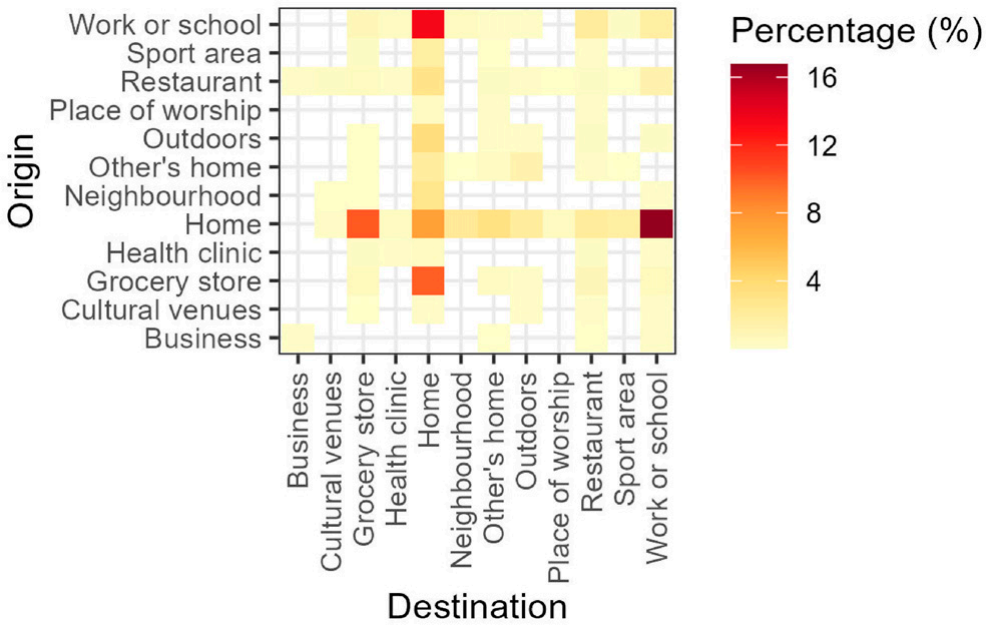
Percentage of walking trips categorized by origin and destination.

{ActiveCA} also enables visual analysis of active travel in Canada using exploratory data analysis techniques. Figure 2 shows walking trips from 2022 through heat maps. This graph uses color gradients to represent the percentage of trips between various origins and destinations, with darker colors indicating higher percentages and lighter colors representing less frequent routes. For conciseness, we omitted the heat maps for the other years analyzed.

In 2022, home location served as a central hub for most trips, with fewer than 10% of journeys not involving it as either a starting point or destination. The most common trip types were from home to work or school (17%) and the reverse, from work or school to home (13%). Notably, 7% of trips began and ended at home, often reflecting leisure activities such as short walks or dog walking. Grocery stores were also a key destination, comprising 10% of trips departing from home.

The {ActiveCA} dataset also includes information on the type of population center in which respondents reside—specifically, whether they live in a CMA, a CA, or outside these areas—as well as the respondent’s province. This information is important, as patterns of active travel often differ between metropolitan and non-metropolitan populations. For example, Table 7 presents the median walking durations by population center type and province for 2022. Overall, respondents living in CMA/CA areas tend to report higher median walking durations compared to those living outside these centers. The most pronounced difference is observed in Nova Scotia: metropolitan residents reported a median walking duration of 30 min, whereas non-metropolitan residents reported a median of only 5 min.

**Table 7.** Differences in walking duration (in minutes) between provinces and population center type.

	Population center type	
Province	CMA/CA	non CMA/CA
Alberta	15	5
British Columbia	15	10
Manitoba	10	10
New Brunswick	5	5
Newfoundland and Labrador	20	10
Nova Scotia	30	5
Ontario	15	15
Prince Edward Island		5
Quebec	15	15
Saskatchewan	10	5

Note. CMA denotes Census Metropolitan Area and CA denotes Census agglomeration.

**Figure 3.**
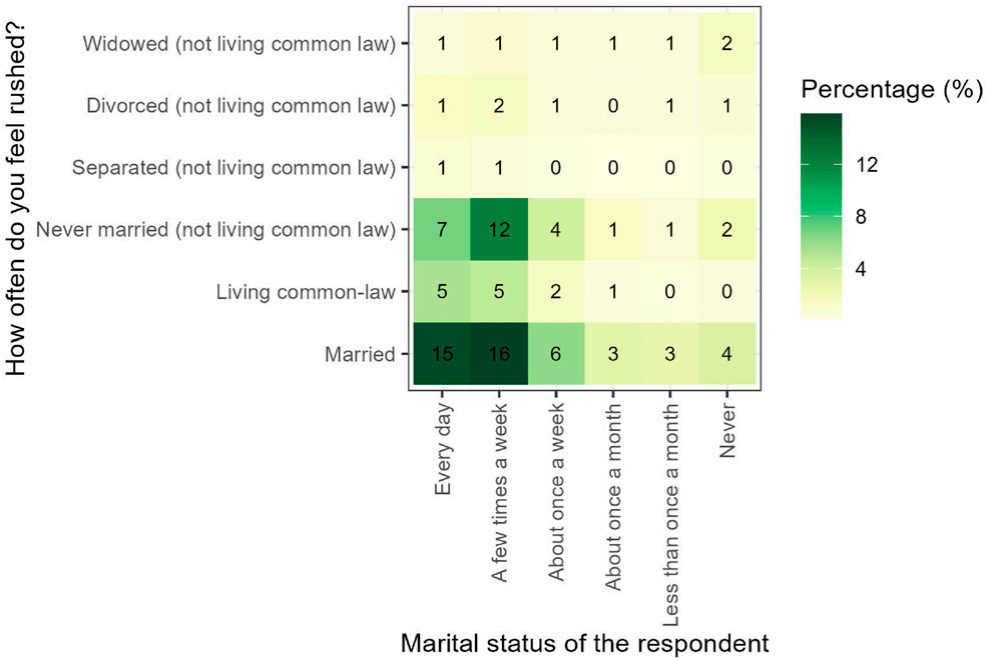
Level of stress among respondents of different marital statuses (2015).

The package also enables obtaining insights from the main processed files. Figure 3 present how the level of stress varied among respondents depending on their marital status in 2022. According to this plot, married respondents reported the highest level of stress, relating to feel stressed every day, with 15% of possible cases.

## Python integration

{ActiveCA} also provides a Jupyter Notebook containing a Python script that demonstrates how to read R data files (.rda) and convert them into Pandas Data Frames. This process allows users to work with and utilize the datasets available in {ActiveCA} within a Python project.

## Concluding remarks

This paper presents {ActiveCA}, an open data product that provides analysis-ready data from Cycles 2 (1986), 7 (1992), 12 (1998), 19 (2005), 24 (2010), 29 (2015), and 34 (2022) of TUS GSSs on active travel in Canada. In the form of an R data package, {ActiveCA} was developed after collecting, cleaning, and processing the survey data, providing information on origins, destinations, and duration of active travel, as well other information.

Although we did not select non-AT episodes, the process for obtaining them is very similar to that used for selecting AT episodes. Researchers interested in non-AT modes can use our framework to guide their methodology, making the small but necessary adjustments. We focused exclusively on AT episodes because the {ActiveCA} package is part of a larger project aimed at analyzing the historical evolution of active travel behavior in Canada.

The value of {ActiveCA} lies in its transparency, accessibility, and ease of use, which facilitates the addition of complementary data sets in the future. R users can seamlessly explore TUS walking and cycling episodes, with the option to suggest enhancements to the package as needed. This article adopts the structure proposed by Soukhov and Páez (2023), whose work provided essential guidance for the creation of this package. Similarly, we aim to contribute to the academic community by promoting transparent research practices that encourage replication and innovation in related fields. We believe that {ActiveCA} will serve as a basis for further research on TUS and for the integration of additional data by the authors or the wider open source community.

## Data Availability

The {ActiveCA} R data package can be found and installed on Github (dias-bruno/ActiveCA).*
